# Modeling and Experimental Study on Characterization of Micromachined Thermal Gas Inertial Sensors

**DOI:** 10.3390/s100908304

**Published:** 2010-09-02

**Authors:** Rong Zhu, Henggao Ding, Yan Su, Yongjun Yang

**Affiliations:** 1 State Key Laboratory of Precision Measurement Technology and Instrument, Department of Precision Instruments and Mechanology, Tsinghua University, Beijing, 100084, China; E-Mail: zrwzwyj@sh163.net; 2 School of Mechanical Engineering, Nanjing University of Science & Technology, Nanjing, China; E-Mail: suyan@mail.njust.edu.cn; 3 The 13th Research Institute of CETC, Shjiazhuang, China; E-Mail: memsyang@sohu.com

**Keywords:** micromachined thermal inertial sensor, heat convection, modeling, nonlinearity

## Abstract

Micromachined thermal gas inertial sensors based on heat convection are novel devices that compared with conventional micromachined inertial sensors offer the advantages of simple structures, easy fabrication, high shock resistance and good reliability by virtue of using a gaseous medium instead of a mechanical proof mass as key moving and sensing elements. This paper presents an analytical modeling for a micromachined thermal gas gyroscope integrated with signal conditioning. A simplified spring-damping model is utilized to characterize the behavior of the sensor. The model relies on the use of the fluid mechanics and heat transfer fundamentals and is validated using experimental data obtained from a test-device and simulation. Furthermore, the nonideal issues of the sensor are addressed from both the theoretical and experimental points of view. The nonlinear behavior demonstrated in experimental measurements is analyzed based on the model. It is concluded that the sources of nonlinearity are mainly attributable to the variable stiffness of the sensor system and the structural asymmetry due to nonideal fabrication.

## 1. Introduction

The development of micromachined inertial sensors has been widely addressed for many years. Typical inertial sensors are based on the movement of a seismic proof mass caused by an inertial quantity. These sensors utilize different sensing principles: capacitive, piezoresistive and piezoelectric measurements [1–3]. Different from these conventional devices, micromachined thermal gas inertial sensors based on heat convection, such as thermal accelerometers [4] and thermal gas gyroscopes [5], offer the advantages of simple structures, easy fabrication, high shock resistance and good reliability due to their use of a gaseous medium instead of a mechanical proof mass as the key moving and sensing elements. The working principle of these thermal inertial sensors is mainly based on the natural convection of gas in a small sealed chamber. In our previous work [5], we demonstrated a low-cost, thermo-fluidic micromachined inertial sensor, the configuration of which consisted of a small silicon etched cavity, a suspended central heater that heated up and lowered the density of the surrounding gas, and four suspended detectors symmetrically placed on two sides of the heater, all of which were assembled and packaged in a hermetic chamber. The proposed sensor could detect single-axis angular rate and dual-axis accelerations. In this paper, we only consider the angular rate detection using the sensor.

A mechanism analysis along with mathematical modeling is an essential part of the required work in the sensor design and sensor optimization processes, especially for an inertial device. An analytical model often helps to understand the behavior of a device and resolve any concurrent problems. For example, an inertial sensor generally has nonlinear problems that usually lower the sensitivity and narrow the working range of the device. In order to get rid of these problems, many researchers have taken great efforts to investigate the nonlinear mechanisms and identify the nonideal sources by modeling [6]. For a thermal gas inertial sensor, systematic modeling is inevitably important for its design and error analysis [7]. However, the modeling in a fluidic and thermal domain is more complicated than in a seismic-mass-based device due to the complexity of multi-physics coupling among electrical, thermal, fluidic, and mechanical properties. Up to now, the corresponding results of modeling in a system level for thermal gas gyroscopes have been rarely reported.

In this paper, theoretical and experimental studies on characterization of a micromachined thermal gas gyroscope are presented. For the first time, a characterization of the sensor incorporating its signal conditioning using a simplified model of a spring-damping system is proposed and experimental verification is demonstrated. The modeling approach relies on the fundamentals of fluid mechanics and heat transfer, in association with empirical techniques. The proposed compact model is effective to handle the complexity of the device optimization. The experimental data are provided from both of model-based simulations and physical measurements using fabricated prototypes. The nonlinear characteristics of the sensor are analyzed based on the model and the nonideal sources are summarized.

## 2. Device Operation and Design

**Figure 1.**
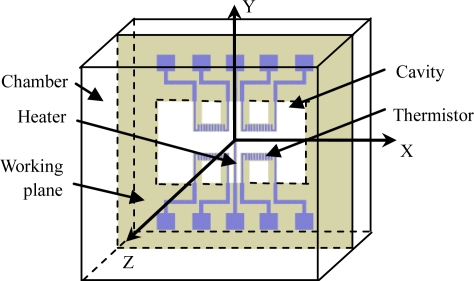
Conceptual design of a thermal gas gyroscope.

**Figure 2.**
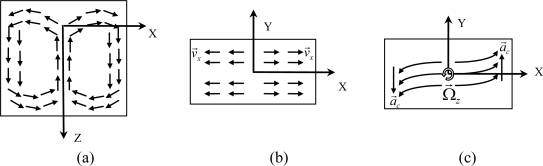
The convection field in region of hermetic chamber driven by heating the central heater under an acceleration along Z-axis. **(a)** The convective flow in the plane of X-Z; **(b)** The flow in the working plane of X-Y; **(c)** the flow deflection due to the Coriolis effect.

**Figure 3.**
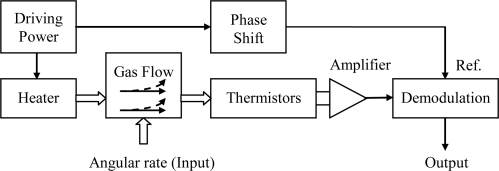
Block diagram of signal transfers in the thermal gas gyroscope.

A conceptual design of a micromachined gas gyroscope is shown in Figure 1. Its convection field in region of hermetic chamber is shown in Figure 2, and the signal transfer and processing strategy are shown in Figure 3.

The working principle of the device is based on the phenomenon of natural convection. A convectional flow is generated by heating the suspended central heater. For instance, when the central heater heats up and acceleration is applied on the direction of the Z-axis, a gas flow is generated in the region of the hermetic chamber and depicted in Figure 2. On the working plane where the detecting thermistors are symmetrically placed, convection flows mainly move along X-axis and are inversely symmetric about the Y-axis. The external inertial rotation Ω⃗*_z_* around the Z-axis will induce a Coriolis acceleration *a⃗_c_* and leads the convective flows on the two sides of the heater to deflect in opposite directions of Y, which can be detected by the distributed detectors (thermistors) in a Wheatstone bridge circuit. Like most vibratory gyroscopes [6], the detection system together with the signal conditioning electronics of the gas gyroscope comprise two orthogonal gaseous oscillators. One of the oscillators, called the primary oscillator or the drive oscillator, is driven by applying an alternating power on the central heater to modulate the convective flow. When the gyroscope rotates about its sensitive axis (*i.e.*, the Z-axis), the Coriolis effect couples the vibration from the primary oscillator to another oscillator in the deflection along the Y-axis, called the secondary oscillator or the sense oscillator. As a result of the Coriolis coupling, the secondary oscillator movement contains the angular rate information, which is the amplitude of the signal modulated around the operating frequency. To obtain the angular rate information, the movement of the secondary oscillator has to be converted into a voltage, and thereafter, be demodulated.

## 3. Modeling

**Figure 4.**
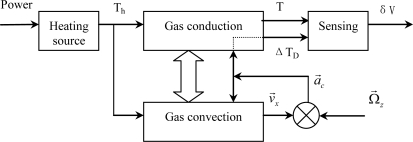
Block diagram of the sensor model.

The entire working process of the sensor consists of multi-physics interactions: electrical-thermal conversion, heat transfer, flow convective movement, and fluid-electrical conversion. A block diagram of the system model, including heating source, gas conduction, gas convection, and sensing, is shown in Figure 4.

Firstly, we consider the heating source. The electric power supplied to the heating resistor is dissipated by heat transfer toward the ambient fluidic medium and also toward the substrate (heating resistor), and which leads to a temperature difference between the heater and ambience. According to the Energy Principle [8], the dynamic process of the heating can be modeled by:

(1) $$\begin{array}{c} C\frac{\partial T_{h}}{\partial t}=P_{h}-{\mathrm{hg}}_{0}\Delta T_{h}\\ \Delta T_{h}=(T_{h}-T_{a}) \end{array}$$

where *T_h_* and *T_a_* refer to the temperatures of the heater and ambience, *C* is the thermal capacity of the heater, *P_h_* is the electrical power, *h* is the heat transfer coefficient, and *g*_0_ is a constant coefficient depending on the geometrical parameters of the heater. According to linear perturbation theory, the heat transfer coefficient *h* can be considered to be constant. Perform Laplace transform to (1), the transfer function of the heating source can be formulated by a first-order model, where *s* represents differential operator:

(2) $$G_{1}(s)=\frac{T_{h}(s)}{P_{h}(s)}=\frac{k_{1}}{1+\tau_{1}s}$$

where τ_1_ = *C/hg*_0_, *k*_1_ = 1*/hg*_0_.

Then, we analyze the process of gas conduction. The gas conduction is the heat conduction. The temperature difference between the heater and external ambience leads to a heat transfer in the gaseous medium in the chamber. According to the heat transfer principle [8], the local temperature *T* at a point in the chamber can be ruled by:

(3) $$\frac{\partial (\rho \mathrm{cT})}{\partial t}=k_{2}\cdot \nabla^{2}(T)$$

where, *ρ*, *c*, and *k*_2_ are the gas density, specific heat, and thermal conductivity, respectively. The vector operator ∇ is defined as $\nabla \equiv i\frac{\partial }{\partial x}+j\frac{\partial }{\partial y}+k\frac{\partial }{\partial z}$. Here we only consider the heat flow within the working plane and define *x* as characteristic dimension for the device. Therefore equation (3) can be reduced to $\frac{\partial (\rho \mathrm{cT})}{\partial t}=k_{2}\cdot \frac{\partial^{2}T}{\partial x^{2}}$. Solving the partial differential equation using a Separation Variable technique [9] together with the boundary conditions *T_h_* at the wall of the heater, we obtain the following first-order transfer relationship:

(4) $$G_{2}(s)=\frac{T(s)}{T_{h}(s)}=\frac{1}{1+\tau_{2}s}$$

where *τ*_2_ = −*ρc*∫∫*T*_0_(*x*)*dx*^2^/(*k*_2_*T*_0_(*x*)), and *T*_0_(*x*) is a normalized shape function of temperature profile.

In the process of gas convection, the gradient pressure is generated by the gradient temperature in terms of the state equation $\rho =\frac{p}{R\cdot T}$, where *p* and *R* are the pressure and gas constant, respectively. According to the Navier-Stokes equation [10], the convection flow velocity *v⃗* of the gas in the chamber is ruled by:

(5) $$\frac{\partial (\rho \vec{v})}{\partial t}=-\nabla (p)+\nabla (\mu \cdot \nabla (\vec{v}))$$

where *μ* is the dynamic viscosity of the gas in the chamber. Solving (5) using the Separation Variable approach and combining the state equation together with the wall condition *v⃗_w_* = 0, we obtain the transfer function between the temperature *T* and the flow velocity *v⃗* of the gas given by a first-order expression:

(6) $$G_{3}(s)=\frac{\vec{v}(s)}{T(s)}=\frac{k_{3}}{1+\tau_{3}s}$$

where *τ*_3_ = −*ρv*_0_(*x*)/(*μ*∇^2^*v*_0_(*x*)), *k*_3_ = −*Rα*_2_∇*T*_0_(*x*)/*T*_0_(*x*), and *v*_0_(*x*) is a normalized shape function of convection flow.

Following the gas momentum equation and Archimedes’s law, an applied acceleration results in a buoyancy force and deforms the temperature profile [8]. When an angular rate Ω⃗*_z_* is applied about the Z-axis, the Coriolis acceleration *a⃗_c_* = 2Ω⃗*_z_* × *v⃗* is generated, which leads to a deformation on the temperature profile that is detected by the thermistors. The temperature deformation has been found to be proportional to the Grashof number *G_r_* determined by a given acceleration [4], which comes a linear relationship between the temperature difference Δ*T_D_* across the thermistor detectors and the given acceleration (here is Coriolis acceleration *a_c_*):

(7) $$\Delta T_{D}\propto G_{r}\;\;\;\mathrm{with}\;\;\;Gr=\frac{a_{c}\rho^{2}\eta T_{h}l^{3}}{\mu^{2}}$$

where η is gas coefficient of expansion, *l* is linear dimension. Considering the governing transient momentum process [8], the above transformation also corresponds to a first-order response:

(8) $$G_{4}(s)=\frac{\Delta T_{D}(s)}{\vec{v}(s)}=\frac{\Omega_{z}k_{4}}{1+\tau_{4}s}$$

where *k*_4_ and *τ*_4_ are constant coefficients depending on thermal and fluidic properties of gas.

The thermistors convert the thermal signals (local temperatures) into the resistance signals of the resistors. Due to thermal inertia of the thermistors, another first-order transfer function should be considered since thermistors have to be in equilibrium with the local temperature of the gas to convert temperature variations into electrical resistance variations. The first-order transfer function represents the signal transfer from the local temperature difference Δ*T_D_* to the temperature difference on the thermistors Δ*T_d_*:

(9) $$G_{5}(s)=\frac{\Delta T_{d}}{\Delta T_{D}}=\frac{1}{1+\tau_{5}s}$$

where *τ*_5_ represents the time constant of the thermal inertia of the thermistors.

Using a Wheatstone bridge circuit, the temperature difference on the detecting thermistors is proportionally converted into a voltage difference *δV* [5]. This process can be formulated by:

(10) $$\delta V=k\prime \cdot \Delta T_{d}$$

where *k*′ is a constant coefficient depending on the parameters of the electronic circuit.

Combining the equations (2), (4), (6), (8), (9), and (10), the entire transfer function from the heating power *P_h_* to the output voltage *δV* can be given by:

(11) $$H(s)=\frac{Y(s)}{X(s)}=k\prime G_{1}(s)G_{2}(s)G_{3}(s)G_{4}(s)G_{5}(s)$$

where *X*(*s*) refers to the Laplace vector of the applied electrical power *P_h_* on the heater, *Y*(*s*) refers to the Laplace vector of the output voltage *δV*. For easing up the analysis for the system and considering the time constant of individual process *G* is generally small value typically in the order of *ms* or *μs*, we ignore the high-order terms in (11) so as to yield a compact simplified spring-damping model formulated by a second-order differential equation:

(12) $$H(s)=\frac{\lambda \Omega_{z}}{s^{2}+\mathrm{cs}+k}$$

where *k* = 1/*τ*″ and *c* = *τ*′/*τ*″ denote equivalent stiffness and damping coefficient, *λ* = *k*′*k*_1_*k*_3_*k*_4_/*τ*″ is a gain representing the sensor sensitivity; $\tau \prime =\sum_{i=1}^{5}\tau_{i}$ and *τ*″ = *τ*_1_*τ*_2_ + *τ*_1_*τ*_3_ + *τ*_1_*τ*_4_ + *τ*_1_*τ*_5_ + *τ*_2_*τ*_3_ + *τ*_2_*τ*_4_ + *τ*_2_*τ*_5_ + *τ*_3_*τ*_4_ + *τ*_3_*τ*_5_ + *τ*_4_*τ*_5_.

In practice, the coefficients *k*, *c*, and *λ* can be identified through experimental calibration. The response function at a frequency *ω* is further modeled in the frequency domain:

(13) $$H(j\omega )=\frac{\lambda \Omega_{z}(j\omega )}{{\left(j\omega \right)}^{2}+c(j\omega )+k}$$

Extract the amplitude and phase of the output response as:

(14) $$\begin{array}{c} |H(j\omega )|=\frac{\lambda \Omega_{z}(\omega )}{\sqrt{{\left(k-\omega^{2}\right)}^{2}+{\left(c\omega \right)}^{2}}}\\ ∠H(j\omega )=-\mathrm{arctg}\frac{c\omega }{k-\omega^{2}}=\theta \end{array}$$

As explained in Section 2, the sensor output signal is detected using a synchronous demodulation technique, which can greatly eliminates disturbances and reduces noise level so as to enhance the accuracy and sensitivity of the sensor. The heating power is modulated at the frequency of *ω*, which leads the corresponding temperature, convection flow, and thermoelectric conversion signals to be the carrier signals at *ω*. The amplitude |*H*| of the output voltage signals is extracted using demodulation, *i.e.*, multiplying the detected signal by a local reference oscillator with the same frequence and phase as the carrier of the detected signal to convert the detected signal (incoming signal) into a dc version. After low-pass filtering, the incoming signal consisting of the carrier at *ω* is retained and others are filtered. For guaranteeing in-phase, the original phase of the local reference oscillator is usually shifted. Define a phase shift Δ*θ*, the normalized demodulation output signal is given by:

(15) $$\begin{array}{c} V_{\mathrm{output}}=|H|\text{cos}(\theta -\Delta \theta )\\ =H_{0}\Omega_{z}\text{cos}(\theta -\Delta \theta ) \end{array}$$

where, $H_{0}=\frac{\lambda }{\sqrt{{\left(k-\omega^{2}\right)}^{2}+{\left(c\omega \right)}^{2}}}$. Ideally, the phase shift Δ*θ* of the reference oscillator needs to be adjusted to be equal to the phase *θ* of the incoming signal for guaranteeing synchrony. As a result the normalized demodulation output is |*H*| = *H*_0_Ω*_z_*. It implifies the ideal output of the sensor is linear with the angular rate Ω*_z_*.

## 4. Nonideal Factors in Sensors

The preceding analyses are based on the assumption of ideal gas and ideal device-structure. However, the practical conditions are complex and in general not ideal. The considered nonideal aspects affecting the device are mainly as follows: inaccurate phase–shift, asymmetrical structure due to unsatisfied fabrication, nonlinear dependence between temperature differences across detectors and Coriolis acceleration.

The first nonideal factor is an improper phase shift in the local reference oscillator due to improper electronic circuits, which will reduce the scale factor of the sensor (*i.e.*, sensitivity) according to (15). Since the phase *θ* of the output response is a function of the driving frequency *ω* according to (14), the compensation-purposed phase shift Δ*θ* of the reference oscillator needs to be carefully adjusted along with the variation of *ω*.

The second nonideal factor affecting the sensor output is structural asymmetry in the chamber, heater, and detectors (*i.e.*, thermistors). Ideally, the suspending heater beam needs to be located in the centre and the chamber needs to be symmetrical in structure in order to generate symmetrical convection flows; the distributed thermistor wires (four thermistors are used in our device) need to be identical and placed symmetrically on two sides of the heater to detect the deflection of the gas flow [5]. However, these ideal symmetry conditions are difficult to realize in a practical fabrication. These structural asymmetries will induce a parasitical term existing in the output signal, and exhibit as a zero offset voltage depending on the fluidic and thermal inertia of the sensor element. Considering this asymmetrical factor, the model in (15) should be modified as follow, which will be proved in the experiments:

(16) $$V_{\mathrm{output}}=H_{0}\cdot [\delta \cdot \text{cos}(\theta \prime -\Delta \theta )+\Omega_{z}\cdot \text{cos}(\theta -\Delta \theta )]$$

where *δ* represents the asymmetrical coefficient, *θ*′ is the phase of the zero-offset output.

The third nonideal source comes from nonlinear dependence of temperature difference across detectors on Coriolis acceleration. A similar nonlinear phenomenon was found in a thermal accelerometer based on heat conduction [11,12], where the sensor output correlation with the temperature is a nonlinear function of the applied acceleration; for a small acceleration there is a linear dependence between temperature and acceleration, whereas with increasing acceleration the non-linearity increases. The nonlinear dependence between the acceleration and temperature difference in our devices behaves as a hardening spring, for large impact forces the spring becomes harder than it does for low impact forces. This nonlinearity is attributed to the gas properties with inconstant viscosity, compressibility, slip boundary or even more complicated effects. Especially in a confined space, the thermal and fluidic properties of the gas are variable with inertia [13]. Therefore, the equivalent stiffness *k* of the system should be a function of the angular rate Ω*_z_*, *i.e.*, *k* = *k*(Ω*_z_*).

## 5. Experimental Study and Analysis

**Figure 5.**
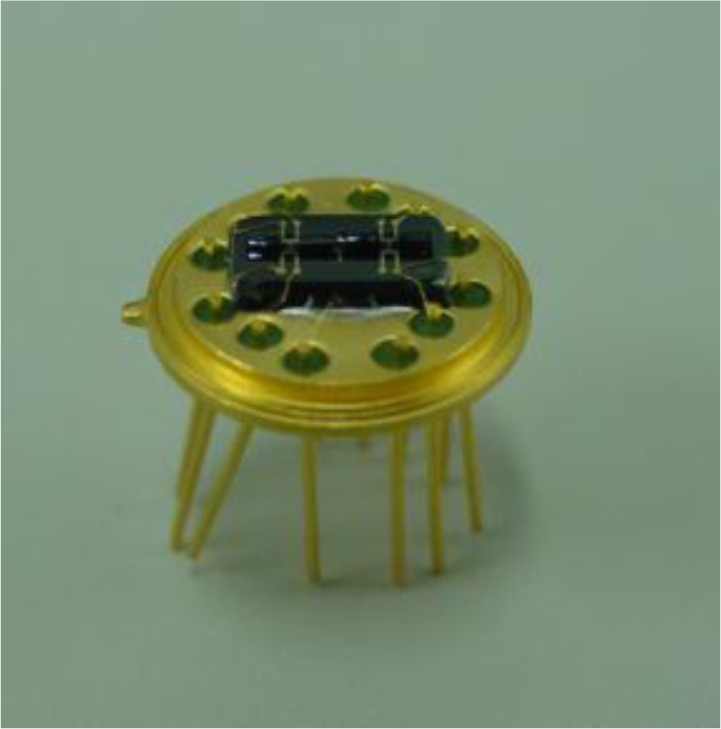
Fabricated sensor prototype without packaging.

To validate the effectiveness of the model established above, we conducted experiments using a device prototype shown in Figure 5, fabricated using micromachining techniques. The detailed fabrication process has been introduced in our previous paper [5]. The sensor was heated by applying an ac power at a given frequency to the heater, and four detectors (*i.e.*, thermistors) in a Wheatstone bridge circuit detected the flow deflection in the chamber that was correlated with the external rotation and exported an output, which was demodulated by a reference signal with the same frequency as the output.

**Figure 6.**
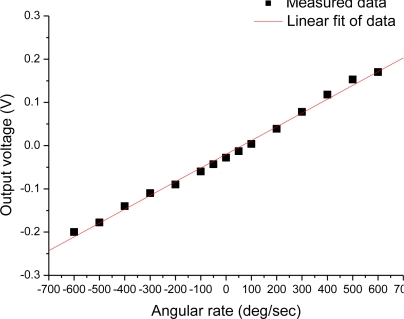
Output voltage of the sensor *versus* the angular rate applied around the Z-axis.

The prepared sensor (device A) was mounted on a controlled rotary table. The Z-axis of the sensor was aligned vertically so that the Earth’s gravity acceleration was applied on the Z-axis of the sensor. The angular rate ranging from −600 deg/sec up to +600 deg/sec was applied around the Z-axis of the sensor. The output voltages of the sensor under a modulation/demodulation frequency of 8 Hz (*i.e.*, the frequency of ac power on the heater) are shown in Figure 6. A near linear relationship between the output voltage and the angular rate was exhibited. However, it is seen that the linearity for small angular rate is better than that for large angular rate, which is consistent with the theoretical analysis.

**Figure 7.**
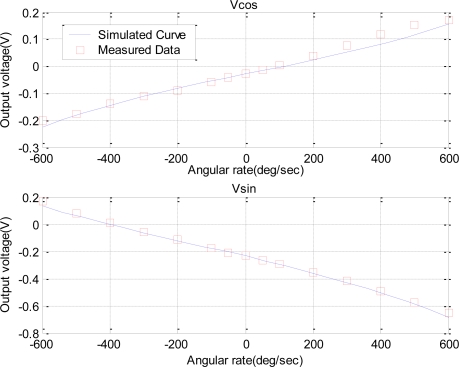
Tow orthogonal components of the output vector *versus* the angular rate in a dual-phase measurement for device A.

To investigate matters of nonlinearity, we further conducted a dual-phase demodulation measurement on the device and used the established model to simulate the output of the sensors. In the dual-phase measurement, two orthogonal reference signals with the same frequency and a phase difference of 90° were used to multiply the detected signal to obtain two orthogonal components of the output vector: *Vcos* and *Vsin*, respectively. According to equation (16), the theoretical formula of *Vcos* and *Vsin* are *H*_0_ ·[*δ* · cos(*θ*′−Δ*θ*) + Ω*_z_* · cos(*θ* − Δ*θ*)] and *H*_0_ · [*δ* · sin(*θ*′−Δ*θ*) + Ω*_z_* · sin(*θ* − Δ*θ*). The simulated outputs based on the theoretical model and the real measured data are compared in Figure 7, which demonstrate a good agreement between the simulated and measured results. The corresponding identification of the model parameters indicated that the equivalent damping coefficient *c* and the gain *λ* were about 12 and 0.62, respectively, the equivalent stiffness *k* increased gradually from 1.72 × 10^3^ to 1.81 × 10^3^ with the increase of the magnitude of angular rate, and an asymmetrical coefficient *δ* was around 350 for device A.

**Figure 8.**
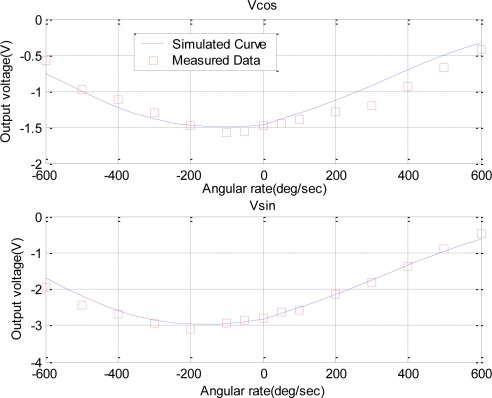
Tow orthogonal components of the output vector *versus* the angular rate in a dual-phase measurement for device B.

To further test the nonlinearity dependence on the stiffness and structural asymmetry, we used another device (device B) with a serious nonlinear feature to conduct experiments. The experimental setup for the device B was same as that for the device A. The dual-phase measurements were used once again in this experiment. Figure 8 demonstrates the measured results and model-based simulation.

**Figure 9.**
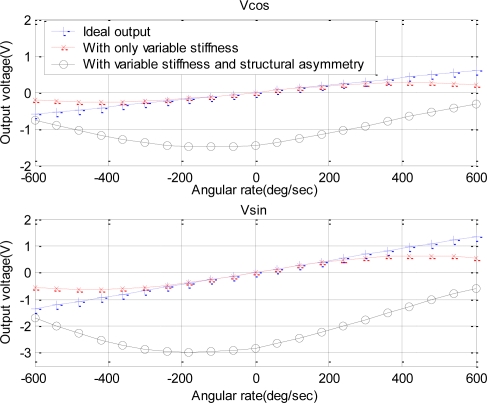
Simulation results of two orthogonal components of the output vector *versus* angular rates in three circumstances: ideal state, with variable stiffness, with variable stiffness and structural asymmetry.

The model parameters were identified by fitting the measurement data. For device B, the equivalent damping coefficient *c* and the gain *λ* were 17 and 4, respectively, the equivalent stiffness *k* varied from 3.95 × 10^3^ up to 6.08 × 10^3^ with the increase of angular rate, and an asymmetrical coefficient *δ* was as large as 1,300. The larger asymmetry induced a serious nonlinearity, and even produced unilateral warp. For identifying the nonlinear sources, we simulated the sensor output under different conditions: with only variable stiffness or with both of variable stiffness and structural asymmetry. The results are shown in Figure 9. It was seen that the variable stiffness contributed to the symmetrical nonlinearity shown as dashed lines with x-marks, and the structural asymmetry contributed to the asymmetric warp shown as solid lines with circle-masks.

**Figure 10.**
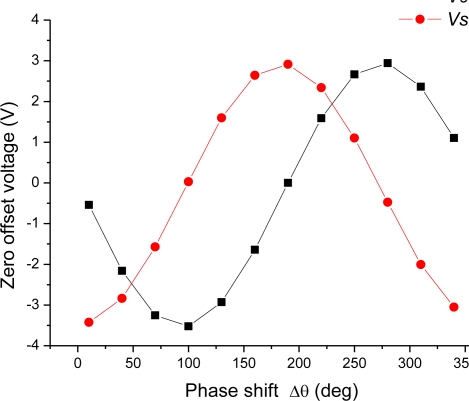
Two orthogonal components of the zero output *versus* the phase shift Δ*θ*.

The structural asymmetry also brings on a zero offset existing in the sensor output as shown in Figure 7 and Figure 8. According to the model (16), the zero offset voltage *V*_0_ = *H*_0_*δ* cos(*θ*′−Δ*θ*), which varies with the phase shift Δ*θ* in cosine law. This dependence between the zero offset and the phase shift was confirmed by an experimental measurement on the device B, in which the phase shift Δ*θ* was changed from 0 deg to 360 deg while the device was kept still. The measured results are shown in Figure 10, where *Vcos* and *Vsin* denote two orthogonal components of the zero offset; theoretically they are *H*_0_*δ* cos(*θ*′−Δ*θ*) and *H*_0_*δ* sin(*θ*′−Δ*θ*), respectively. Figure 10 indicates that the measured data follow cosine and sine function of *Vcos* and *Vsin* very well.

From the preceding measurements and analyses, it is seen that the model established in the paper can characterize well the performance of the sensor, and is feasible to be used for the optimal design and device improvement. It is also seen that the structural symmetry in the device is crucial for the linearity. The fabrication needs to be improved to amend structural asymmetry for eliminating the nonlinearity of the sensor. Besides the linearity of the sensors, we also tested noise limited resolution of the angular rate for the sensors. We found the noise densities of the sensors were around $1\;\text{deg}/s/\sqrt{\mathrm{Hz}}$.

## 6. Conclusions

A mathematical model (simplified as a spring-damping system) is established for a micromachined thermal gas gyroscope based on convection heat transfer to characterize multi-physics interaction processes: electrical-thermal conversion, convection heat transfer, flow convective activity and fluid-electrical conversion. A signal detection process using a modulation/demodulation technique is considered in the modeling, where the heating power is modulated at a given frequency and the angular rate is extracted by demodulation and a low-pass filter. The established model is validated by comparing the simulated results with the real measured data from dual-phase measurements. The theoretical and experimental studies reveal that the nonideal effects in the device are mainly attributable to the structural asymmetry and the variable stiffness of the system; the linearity of the sensor can be improved via amending the structural asymmetry in fabrication.
